# Primary versus deferred ureteroscopy for the management of obstructive anuria secondary to ureteric urolithiasis in children: a prospective randomized study

**DOI:** 10.1007/s00240-022-01389-0

**Published:** 2022-12-02

**Authors:** Mostafa AbdelRazek, Atef Fathi, Mostafa Mohamed, Mohammad S. Abdel-Kader

**Affiliations:** Department of Urology, Qena University Hospital, South Valley University, Qena, Egypt

**Keywords:** Ureteral stones, Deferred ureteroscopy, Anuria

## Abstract

To compare the role of primary and deferred ureteroscopy (URS) in the management of obstructive anuria secondary to ureteric urolithiasis in pediatric patients. This prospective randomized study included 120 children aged ≤ 12 years who presented with obstructive anuria secondary to ureteric urolithiasis between March 2019 and January 2021. The children were subdivided into group A, which included children who had undergone primary URS without pre-stenting, and group B, which included children who had undergone URS after ureteric stenting. All children were clinically compensated and sepsis-free. Patients with underlying urological structural abnormalities were excluded. The operative time, improvement of renal functions, stone-free rate, and complications were compared between the two groups. At the 1-month follow-up, urine analysis; kidney, ureter, and bladder radiography; and ultrasonography were performed. The patient characteristics of both groups did not show any significant difference. Primary URS had failed in ten children (16.6%) in group A. Moreover, failure of stenting was noted in six patients (11%) in group B. The mean operative time for group B was significantly lower than that for group A (*p* ≤ 0.001). The stone-free rate was significantly higher in group B (*p* ≤ 0.001). The rate of overall complications was higher in group A. Deferred URS is preferable over primary URS in the management of obstructive anuria secondary to ureteric urolithiasis”. In children because of the lower need for ureteric dilatation, higher stone- free rate, shorter procedure time, and lower complication rate.

## Introduction

The rate of urolithiasis in the pediatric population has been increasing daily and causes significant morbidity [1, 2]. Changes in nutritional habits, climate, and environmental factors are some of the possible reasons for the increasing incidence of urolithiasis in the pediatric population [3]. It may be complicated by obstructive anuria and acute renal injury (ARI). In this condition, urgent urinary drainage, using a percutaneous nephrostomy (PCN) tube or retrograde ureteric stenting, is the standard method of care [4, 5].

The hazards of anuria are related to electrolyte imbalance, particularly hyperkalemia, which leads to cardiac arrhythmia and sudden death. Thus, urgent intervention is required [6].

Ureteroscopic management of ureteral obstruction in children was a challenge to the urologists, because relatively large instruments are used in smaller anatomy. However, with the improvement in techniques of lithotripsy and the development of small-diameter endoscopes, endoscopic management has become the main technique for treating urinary stones in children [7, 8].

Secondary ureteroscopy (URS), after initial ureteric stenting, is currently an emerging treatment for ureteric stones in children and adults. The resulting passive dilatation of the ureter facilitates URS and stone manipulation [9].

Primary URS for obstructive anuria secondary to ureteric urolithiasis is to do immediate URS, disintegration and extraction of the stone while deferred URS referred to delayed URS after initial ureteral stenting or percutaneous drainage of the Kidney.

Obstructive anuria secondary to ureteric urolithiasis could be managed by primary or deferred URS. The standard management is deferred URS (initial ureteral stenting or percutaneous drainage). However, the development and improvement of the ureteroscopes and lithotripters favored primary URS [10].

This study aimed to evaluate the role of primary and deferred URS in the management of obstructive anuria secondary to ureteric urolithiasis in pediatric patients.

## Patients and methods

This prospective randomized clinical study included 120 children presenting with obstructive anuria secondary to ureteric urolithiasis at our institution between March 2019 and January 2021. The children were subdivided into two groups depending on the timing of URS: group A included children who had undergone primary URS without pre-stenting, while group B included children who had undergone URS after ureteric stenting using a 4.8–6 Fr JJ stent. The study included children aged ≤12 years, clinically compensated, and sepsis-free. Children with underlying structural urological abnormalities or who were clinically ill were excluded from the study. The data (patients’ age; sex; and stone laterality, and size) were collected after obtaining study approval from the ethics committee of our institution URO016-1-185. The study was registered with ClinicalTrials.gov (identifier no. NCT04980079). Before endoscopic management, informed written consent was obtained from parents of all the children included in the study.

The size and level of obstructing stones and degree of obstruction were determined by abdominal ultrasonography, plain urinary tract imaging, and non-contrast computed tomography. Postoperatively, the urine volume and serum creatinine and electrolyte levels were monitored daily until they returned to normal levels. All patients were observed for clinical and laboratory value improvement.

All URS procedures were performed under general anesthesia using a 6-Fr semi-rigid ureteroscope with parenteral antibiotics administered before the procedure. A holmium laser was used for lithotripsy under fluoroscopic guidance. All stones were fragmented using the laser until they became small enough (compared to the diameter of the guide wire or that of the ureteric lumen) to be removed with graspers or passed spontaneously. Complete stone clearance was defined as absence of stone fragments on endoscopic visualization and imaging, while clinically insignificant fragments were defined as stone fragments < mm. Deferred URS was performed within 15 days of ureteral drainage using 4.8–6 Fr JJ stents or 6–8 Fr PCN tubes after the improvement of the renal function, and anuria was corrected.

The operative time, improvement of renal functions, stone-free rate, complications, and number of interventions to reach the stone-free status were compared between the two groups. At the 1-month follow-up, urine analysis; kidney, ureter and bladder radiography for radiopaque stones; and abdominal ultrasonography were performed to assess the children for urinary tract infection (UTI), residual stone fragments, and renal back pressure.

Calculation of the sample size using a sample size calculator software for 95% power of the study to detect a difference of 10% between the groups regarding SFR and overall complication indicated the need for 30 patients in each group. Statistical analysis was conducted using SPSS^®^ version 21. The patient and stone criteria, procedure details, outcome, and complications of both the groups were compared. The variables that affect preoperative and postoperative conditions were compared using the Pearson chi-square or Student *t* test, as appropriate. A *p* value of <0.05 was considered statistically significant.

## Results

This study included 120 children with obstructive anuria secondary to ureteric urolithiasis who presented to our clinics. They were divided into 2 groups. Group A (60 patients) underwent primary URS and group B (60 patients) underwent deferred URS. The patient characteristics of both groups are presented in Table 1. There was no significant statistical difference between the two groups. Primary URS was conducted in 112 renal units (RU), while deferred URS was conducted in 108 RUs.

**Table 1:** Characteristics of children with calcular anuria who underwent either primary or deferred ureteroscopy

	Group A (primary URS) *N* = 60	Group B (deferred URS) *N* = 60	*p* value
Age (years)	6.93 ± 2.9	6.34 ± 3.4	0.35
Males, *n* (%)	36 (60)	40 (66.7)	0.62
Female, *n* (%)	24 (40)	20 (33.3)	
Site of stones, *n* (%)			
Proximal ureter	12 (20)	10 (16.7)	0.71
Middle ureter	14 (23.3)	18 (30)	
Distal ureter	34 (56.7)	32 (53.3)	
Laterality, *n* (%) 0.13			
Single function kidney	8 (13.3)	12 (20)	
Bilateral	52 (86.7)	48 (80)	
Stone size (mm)	13.5 ± 5.3	14.2 ± 4.6	0.19
Creatinine level on presentation (mg/dL)	3.2 ± 1.09	3.4 ± 1.06	0.61

Data are presented as mean ± SD

*URS* ureteroscopy

*Significant *p* value ≤ 0.05

Primary URS failed in ten children (16.6%) in group A due to ureteral kinks. Four patients (solitary kidney, four RUSs) had proximal ureteric stones, while 6 patients (bilateral obstruction, 12 RUSs) had tight ureters. All of these patients were managed by deferred URS after stent insertion. Moreover, stenting failed in 6 patients (11%, 12 RUSs) in group B due to undetectable ureteric orifices; so, we inserted a unilateral PCN tube in these children and planned for a secondary URS after the improvement of the renal function.

Term “tight ureter” has recently been reported in the literature. It describes a condition where the ureter does not respond to dilatation during URS, resulting in marked resistance during advancement of the ureteroscope. Therefore, serious complications may occur during URS. Thus, passive dilatation of a tight ureter before URS is preferable [12].

**Fig. 1:**
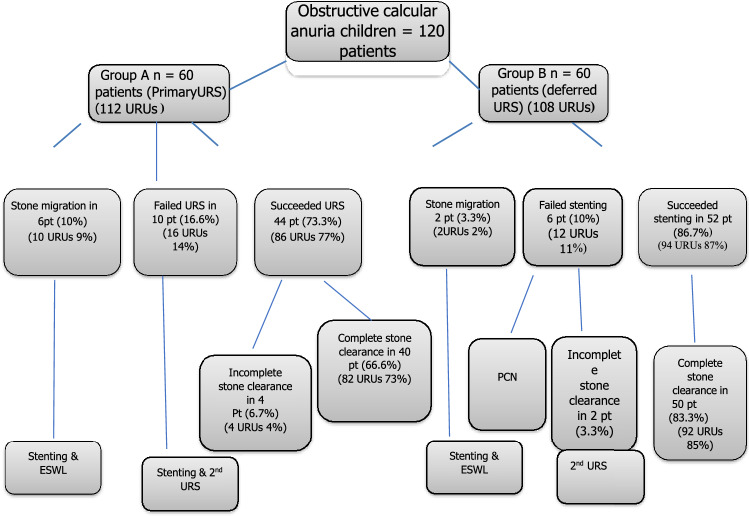
Patient management algorithm. *URS* ureteroscopy; *PCN* percutaneous nephrostomy; *ESWL* extracorporeal shock wave lithotripsy; *URU* ureterorenal unit

Upward migration of stones occurred in three patients (5%, five RUSs) in group A and one patient (1.6%, two RUSs) in group B. All were managed by stenting and extracorporeal shock wave lithotripsy (ESWL). Six children in both groups had incomplete stone clearance (unilateral in each of them, 6 RUSs, 4% in group A and 2% in group B). All were managed by stenting and subsequently subjected to another session of URS. They received anticholinergic treatment to reduce bladder spasm until the JJ stent was removed. The patient’s management plan and stone clearance are shown in Fig. 1.

**Fig. 2:**
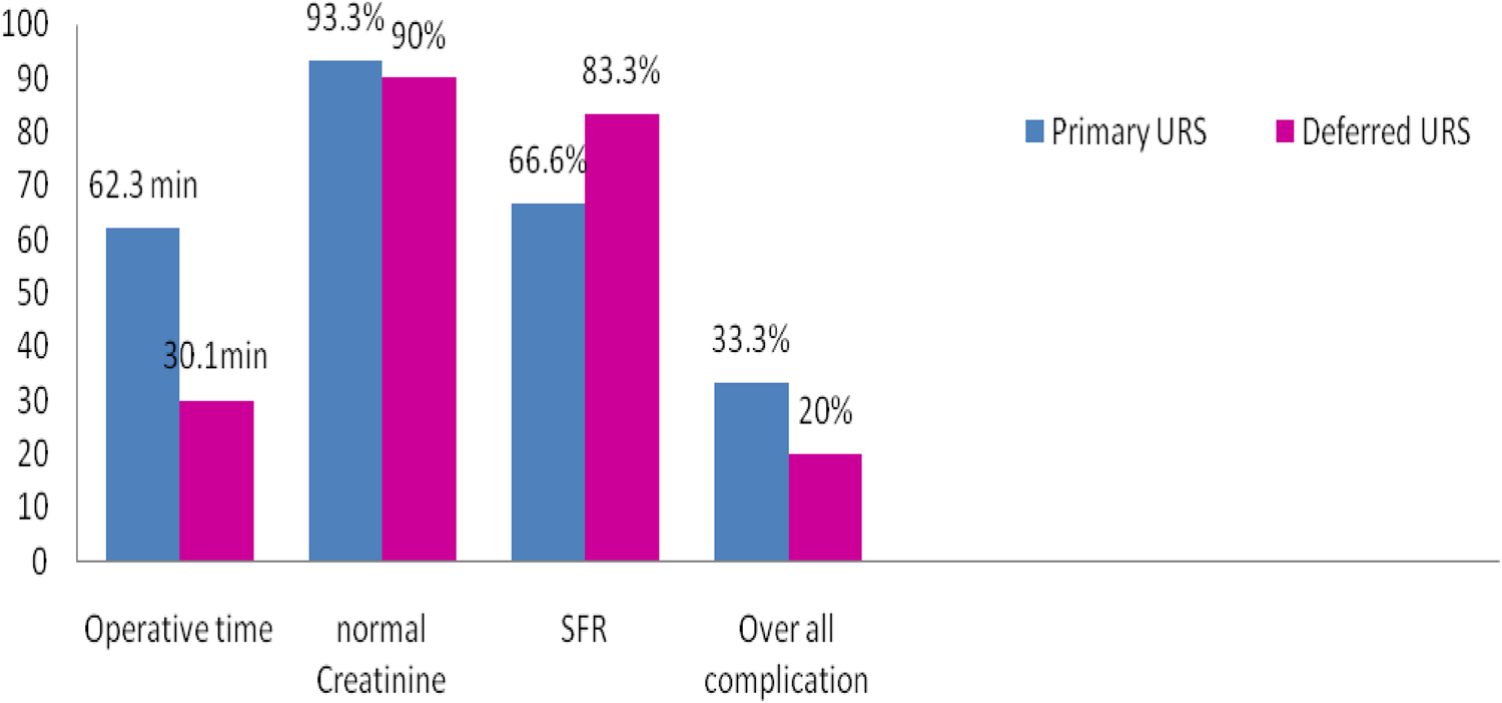
Difference in study outcomes between primary and deferred URS

The mean operative time in group B was significantly lower than that in group A 30.1 ± 8.4 Vs. 62.3 ± 13.4 min (*p* ≤ 0.001). There was no significant difference in the number of patients with restored normal renal function within 1-month follow-up (56 children in group A compared to 54 children in group B, *p* ≤ 0.44) between the two groups. The stone-free rate was significantly higher in group B (*p* ≤ 0.001) (Table 2 and Fig. 2).

**Table 2:** Patient outcomes

	Group A (primary URS) *N* = 60	Group B (deferred URS) *N* = 60	*p* value
Mean operative time (min)	62.3 ± 13.4 min	30.1 ± 8.4 min	0.001^*^
Patients who achieved a normal creatinine level (1 month follow-up), *n* (%)	56 (93.3%)	54 (90%)	0.44
Period to normal creatinine level (days)	2.30 ± 0.73	2.26 ± 0.77	0.17
Stone free, *n* (%)	40 (66.6%)	50 (83.3%)	0.001*
Operative time of first procedure (ureteric stenting)	–	14 ± 5 min	

Data are presented as mean ± SD

*URS *ureteroscopy

*Highly significant *p* value ≤ 0.001

The rate of overall complications was higher in group A, with no statistically significant difference between the two groups (*p* = 0.09). Complications were identified in 14 patients in group A (16.6%) and 9 patients (10%) in group B. All complications are presented in Table 3. All complications were deemed minor (Clavien–Dindo I). Three patients in group A had mucosal injury or marked edema at the end of the maneuver, with no statistically significant difference between the two groups for hematuria, febrile UTI, and postoperative polyuria (Table 3 and Fig. 2).

**Table 3:** Complications

	Group A (primary URS) *N* = 60	Group B (deferred URS) *N* = 60	*p* value
Complications *n* (%)			
Stone migration	3 (5%)	1 (1.6%)	0.13
Mucosal injury	3 (5%)	0 (0)	0.32
Hematuria	4 (6.6%)	2 (3.3%)	0.62
Febrile UTI	1 (1.6%)	3 (5%)	0.42
Postoperative polyuria	3 (5%)	3 (5%)	1.0
Total	14 (16.6%)	9 (10%)	0.09

*URS* ureteroscopy; *UTI* urinary tract infection

*Significant *p* value ≤ 0.05

**Highly significant *p* value ≤ 0.001

## Discussion

The management of pediatric urolithiasis is challenging, especially in the presence of anuria and ARI. The kidney is characterized by its ability to recover from almost complete loss of function. Most cases of ARI are reversible, albeit subclinical minimal defects in tubular and glomerular functions [11].

Ureteral stenting or nephrostomy drainage is the standard treatment for obstructive anuria associated with ureteral-stone obstruction. ESWL is not a method of choice in such cases because of obstruction and poor renal function that leads to poor stone fragmentation and even lower clearance rates [13].

Despite all the challenges involved, URS has now become a part of the endoscopic armamentarium of the pediatric urologist, even in children as young as 18 months [14]. Ureteral-stone migration and retrograde intrarenal disintegration were prevented by improvement of URS skills and techniques, development of small-caliber ureteroscopes, and use of flexible ureteroscopes [15, 16].

This study reported the management of obstructive anuria due to ureteric stones in children aged ˂12 years. In these patients, rapid urologic interventions may occur, by primary URS, PCN, or ureteric stent, to allow urinary drainage and prevent irreversible renal damage.

With regard to primary URS, routine dilatation of the ureteric orifice and the intramural part was performed, with gradual dilatation using ureteric dilators, which was found to be less traumatic compared to dilatation using a ureteroscope. This concept is in agreement with the findings of Minevich et al., who reported that gradual dilatation using ureteric dilators was preferable and less traumatic than other methods [17].

The true incidence of vesicoureteric reflux (VUR) in children following URS after ureteric dilatation versus URS without dilatation remains unknown. Most studies revealed that post URS, VUR incidence is of low grade and resolves spontaneously [18].

Some authors reported that bilateral same-session URS is an effective and safe procedure in the management of bilateral ureteric stones, but upper ureteric, large, and impacted stones carry the highest risk of unsuccessful results [19]. Previous studies reported that bilateral emergency same-session URS is a good choice to reduce hospital stay, prevent multiple anesthesia, and decrease the costs for adult patients with obstructive anuria secondary to ureteric urolithiasis and ARI [19, 20]. Another study reported that bilateral emergency same-session URS may be associated with a higher morbidity rate [21]. In the present study, same-session URS was performed in 42 patients in group A (84 RUSs). We reported failed primary URS in 10 children (16.6%, 8 RUS) in group A due to ureteric kinks in 4 patients with proximal ureteric stones and 6 patients with tight ureters, which were managed by JJ stenting and deferred URS.

In the present study, no children with secondary URS required any further dilatation, as the ureter was passively dilated with a ureteric stent. This may explain the better results in group B. Conversely, 90% of children (54/60) with primary URS (group A) required dilatation.

Although primary URS aims at attacking the stones with the least number of procedures, initial failure of primary URS (16.6%), incomplete stone clearance (6.6%), and postoperative stenting increase the number of procedures being performed. Therefore, its advantage over secondary URS is lost.

In the present study, stone clearance was achieved in 66.6%% and 83.3%% of the children (primary and secondary URS, respectively), which is comparable to the clearance rates of previous studies, ranging from 77 to 100% [17].

Our study showed that deferred URS (JJ stenting) had formed a part of definitive management of stones, with 83% complete stone clearance, shorter mean operative time (30 vs 62.3 min), and low complication rate (10 vs 16.6%); hence, this confirms the importance of stent placement in providing not only urinary drainage but also easier subsequent ureteric manipulation but we should notice the operative time taken during first procedure (ureteric stenting) in deferred URS group which was (14 ± 5 min) and the quality of life of children entering operative theater for two times. Our results are similar to those of a retrospective study by Elgammal et al. who reported that secondary URS in 24 children had better stone clearance, significantly lower need for ureteric dilatation, and shorter intervention time than primary URS in 42 children aged ˂12 years [17].

The current study limitations are the relatively small sample size with limited statistical power and short follow-up time; therefore, further study with a large sample size and long-term follow-up is recommended another limitation is that the children quality of life during both procedures not estimated so we recommend in future studies to be estimated.

## Conclusions

Deferred URS is preferable over primary URS in the pediatric population, especially for the management of obstructive anuria secondary to ureteric urolithiasis in pediatric patients compared to primary URS, because of a significantly lower need for ureteric dilatation, higher stone-free rate, shorter procedure time, and lower complication rate.

## Data Availability

All data generated or analysed during this study are included in this published article tables and The datasets generated during and/or analysed during the current study are available from the corresponding author on reasonable request.
